# Speckle tracking for cardiac MRI in patients pre and post dilation and stent implantation of aortic coarctation

**DOI:** 10.1186/1532-429X-14-S1-P125

**Published:** 2012-02-01

**Authors:** Dirk Lossnitzer, Hannah Bellsham-Revell, Aaron Bell, Andreas Schuster, Tarique Hussain, Rene M Botnar, Reza Razavi, Gerald F Greil

**Affiliations:** 1 Department for Imaging Sciences, King's College London, London, UK

## Background

Current assessment techniques for regional contractility require cardiac magnetic resonance-tagged or strain encoded imaging sequences and their analysis is complex. A new steady-state free precession cine sequence (SSFP) based technique called feature tracking can readily be performed on standard SSFP sequences. To test the feasibility of feature tracking in patients with coarctation of the aorta (CoA) and to quantify regional contractility patterns pre and post percutaneous dilation and stent implantation in the region of CoA.

## Methods

6 consecutive male patients (mean age 15,7 years, range 8 to 25 years) with CoA underwent cardiac MRI pre and 1 to 2 years post stent implantation in a 1,5 Tesla Achieva MR system (Philips, The Nederlands) using a 5 element cardiac coil. 3 conventional SSFP cine short axis slices (apical, midventricular and basal orientation) were analyzed for peak myocardial radial strain, peak myocardial circumferential strain and fractional area change dA/dt (FAC) pre and post catheter intervention using Feature Tracking software (TOMTEC, Germany). Comparison between groups were calculated using a general linear model for repeated measurements (PASW Statistics 18.0, SPSS Inc. USA).

## Results

**Figure 1:**
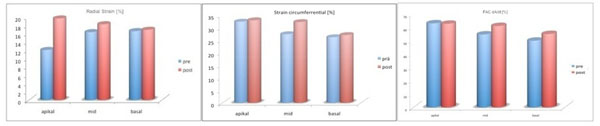
Comparisons of radial strain (left), circumferential strain (middle) and FAC dA/dt (right) pre and post intervention within the apical, midventricular and basal myocardial segments.

**Figure 2:**
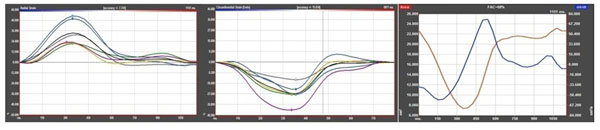
Examples of measurement of radial strain (left), circumferential strain (middle) and FAC dA/dt (right).

Patients showed a post-procedural significant increase of myocardial radial strain (p=0.05) with the highest extent within the apical myocardial segments (Fig.1). Circumferential strain (p=0.55) as well as dA/dt FAC (p=0.52) showed an increase but did not reach the level of statistical significance probably due to the small study group (Fig.1). Measurements could be completed in all patients (Fig.2).

## Conclusions

Feature tracking is a new and promising technique for the assessment of regional contractility using robust, standard SSFP images and can therefore be performed without the need of additional imaging sequences or even retrospectively. Measurements in our study group showed as significant increase of radial myocardial contractility after stent implantation. To verify the results of this pilot study an adjacent trial including more patients as well as a healthy control group is required.

## Funding

None.

